# Design and preparation of core–shell AP@HNS composites with high safety and excellent thermal decomposition performance

**DOI:** 10.1039/d2ra01871c

**Published:** 2022-05-19

**Authors:** Wenxiang Li, Ping Ye, Changping Guo, Wenkun Zhu, Dayong Jin

**Affiliations:** Xi'an Modern Chemistry Research Institute Xi'an 710065 PR China 13572590838@163.com; Sichuan Co-Innovation Center for New Energetic Materials, Southwest University of Science and Technology (SWUST) Mianyang 621010 PR China guochangping001@163.com

## Abstract

Solid propellants with high safety, excellent thermal decomposition, and green performance are hot and difficult research areas in aerospace. In this paper, AP@HNS (hexanitrostilbene) composites with core–shell structure were designed and prepared by an ultrasound-assisted method using polyurethane for the interfacial modification of ammonium perchlorate (AP). The results show that the AP@HNS composites have a complete and dense shell structure when the nano-HNS content of the shell layer is 15% or more, the synergistic decomposition effect between HNS and AP can advance the high-temperature decomposition peak of AP by 102.4 °C and increase the apparent heat release 2.55 times to 1388 J g^−1^, and HNS improves the energy of AP while reducing environmental pollution. The safety performance test shows that the nano-HNS with 15% mass content can increase the composite characteristic drop height *H*_50_ to 32 cm and reduce the frictional susceptibility explosion probability to 77% (the *H*_50_ of AP is 27 cm and the frictional susceptibility explosion probability is 95%). The insensitive shell layer HNS significantly improves the safety performance of AP through barrier and buffering effects. This technology is expected to provide new ideas for designing and preparing solid propellants with high energy, low susceptibility, and excellent thermal decomposition performance.

## Abstract 2

AP@HNS composites with core–shell structure were designed and prepared. This technology is expected to provide new ideas for designing and preparing solid propellants with high energy, low susceptibility, and excellent thermal decomposition performance.

## Graphical abstract

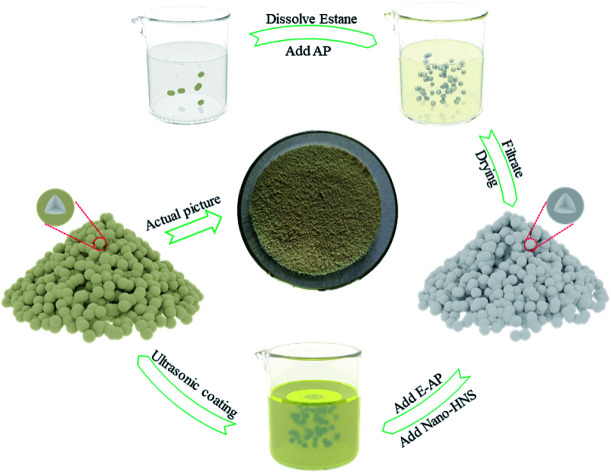

## 1. Introduction

The level of aerospace technology is an essential indicator of a country's military level, and solid propellants provide the necessary energy and power for its operations.^1^ Ammonium perchlorate (AP) is a common oxidizer and the main component of composite solid propellant, which provides the required oxygen for the combustion of composite solid propellant. Its thermal decomposition characteristics are closely related to the combustion behavior of composite solid propellants.^2^ Specifically, reducing the decomposition temperature of AP can effectively reduce the ignition delay of solid propellant, and increase the combustion stability and combustion rate.^3,4^ In recent decades, the thermal decomposition performance of AP has been improved mainly by adding transition metals or their oxides as catalysts, such as porous β-MnO_2_,^5^ nano-Co_3_O_4_,^6^ and nano-ZnO.^7,8^ Generally, high decomposition temperature and heat release are two crucial criteria for evaluating the thermal decomposition performance of an AP compound. However, modern composite propellants need to have higher performance with the enhancement of aerospace requirements. Safety performance and green environment protection have also become critical requirements for developing composite solid propellants.^9–11^

However, using transition metal oxides as catalysts in the formation of smoke after combustion is detrimental to missile operations on the battlefield and pollutes the environment. In addition, the current research on AP has paid little attention to its safety performance. According to the literature, it is reported that transition metals or their oxides are added directly to AP formulations as catalysts to lower the decomposition temperature of AP by reducing its decomposition energy barrier. However, at the same time, the reduction of energy barriers will make AP more prone to safety accidents when subjected to accidental impact or friction, which is unacceptable.^12,13^ Therefore, it is crucial for the study of AP to reduce the high-temperature decomposition peak of AP and increase its heat release while ensuring adequate safety during storage, transportation, and use. In this way, we can reduce the generation of heavy metal fumes and avoid pollution to the environment, which poses a significant challenge to our work.

High-energy blunt explosives have been a hot topic of interest in the field of energy-containing materials. They are often used as modified materials to enhance the safety performance of high-sensitivity explosives. According to the literature, some explosives have a unique synergistic effect with AP, which can effectively improve the thermal decomposition performance of AP and reduce the high-temperature decomposition temperature. At the same time, explosives as energy-containing materials do not reduce the energy of the system compared with metal catalysts, which intuitively shows an increase in the exothermic heat of AP.^14,15^ This unique synergistic effect was also confirmed by the core–shell AP@TATB composites prepared by our group in previous studies. However, its effect on reducing the decomposition temperature of AP is limited because the decomposition temperature of TATB is about 370 °C.^16^ In order to further reduce the decomposition temperature of AP, a lower decomposition temperature explosive, hexanitrostilbene (HNS), was used in this study. A high-energy blunt explosive with good thermal stability performance, capable of an apparent heat release of up to 1712 J g^−1^. Meanwhile, HNS comprises four elements, C, H, O, and N, avoiding transition metals in the catalyst, effectively reducing smoke and pollution.^17,18^ Therefore, this study designed and prepared an AP@HNS composites with a core–shell structure with high safety performance and decreased decomposition temperature.^19–21^

## 2. Experimental section

### 2.1. Materials

Ammonium perchlorate (AP) was purchased from Aladdin Industrial Corporation (Shanghai, China). Hexanitrostilbene (HNS) was obtained from China Academy of Engineering Physics (Mianyang, China). Polyurethane (estane), dichloroethane and anhydrous ethanol were purchased from Mianyang City Letter Jie Trade Co., Ltd., China.

### 2.2. Preparation of nuclear shell AP@HNS

**Fig. 1:**
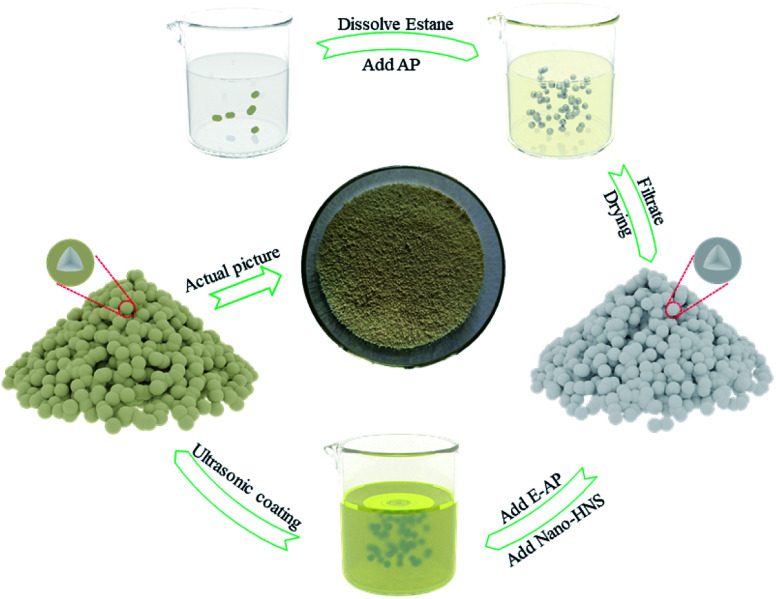
The preparation process of core–shell AP@HNS composites.

This study designed a feasible scheme to prepare AP@HNS composites with core–shell structures. A complete and homogeneous core–shell structure was prepared by ultrasonic shaking and adjusting the ratio of HNS. Fig. 1 shows the flow diagram of the preparation of AP@HNS composites, and the specific preparation steps are as follows.

Firstly, 0.05 g of estane was weighed into a beaker, and 50 mL of dichloroethane was added. Estane was dissolved into dichloroethane by magnetic stirring in a constant-temperature water bath at 50 °C, and then 10 g of AP particles were adding to the beaker. Estane was finally modified on the surface of AP particles after 30 min. After being filtered and dried, the prepared E-AP was added to the appropriate amount of anhydrous ethanol and dispersed by ultrasonic shaking. The nano-HNS was added in a particular proportion and continued to be ultrasonicated for 10 min so that the nano-HNS could be uniformly coated on the surface of the AP particles, in which different proportions of HNS accounted for 2%, 5%, 10%, 15% and 20% of the total mass (AP and HNS), respectively.

### 2.3. Characterization of core–shell AP@HNS

X-ray diffractometer, model X'Pert Pro, with Cu Kα target, scanning range of 10–80°, was used to obtain XRD patterns to characterize the crystal structure of the analyzed samples, tungsten scanning electron microscope, model EVO18, at 15 kV, was used to obtain SEM images to observe the microscopic morphology and particle size of the samples. X-ray photoelectron spectrometer, model K ALPHA+, using Al Kα source at 12 kV, was used to obtain N 1s spectra to analyze the surface structure of the sample, synchrotron thermal analyzer, model STA-449-F3, with 1.0 mg sample, aluminum crucible, nitrogen atmosphere, temperature rise rate 10 K min^−1^, test range 30–500 °C, was used to obtain DSC curves of the sample to analyze the thermal decomposition properties and exotherm. The impact susceptibility test was performed by GJB-772A-97 test method 601.2, and the impact susceptibility data were obtained by dropping the hammer at a mass of 10 kg and sampling 50 mg for 30 times. The friction susceptibility test was performed by GJB-772A-97 test method 602.1, and the pendulum mass was 1.5 kg and sampling 50 mg for 30 times to obtain the explosion probability.

## 3. Results and discussion

### 3.1. The crystal structure of the AP@HNS composites

**Fig. 2:**
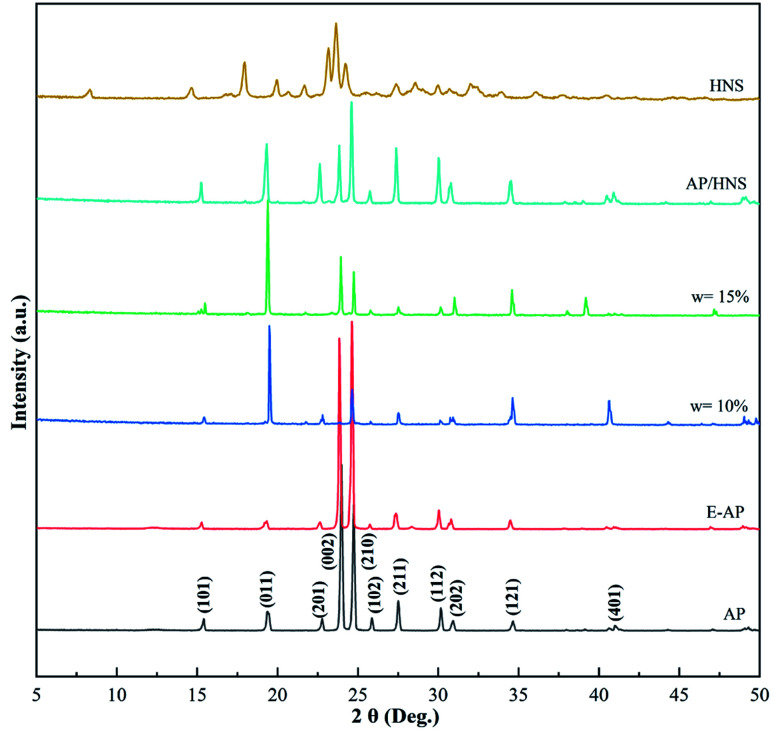
XRD spectra of AP, E-AP, core–shell AP@HNS with 10% and 15% HNS, mechanically mixed AP/HNS with 10% HNS, and HNS.

In order to investigate the effect of HNS on the crystalline phases of AP under the preparation conditions, XRD characterization of raw AP, E-AP, core–shell AP@HNS with 10% and 15% HNS, mechanically mixed AP/HNS with 10% HNS, and pure HNS was carried out in this paper. The results are shown in Fig. 2. The characteristic peaks of AP can be seen in the Fig. 2 at 15.4°, 19.4°, 22.8°, 23.9°, 24.7°, 25.8°, 27.5°, 30.1°, 30.9°, 34.6°, 34.6°, 30.9°, 34.6°, and 41.0°, which are in perfect agreement with the crystal plane on the standard card AP (JCPDS08-0451). The modified AP by estane is highly consistent with the diffraction peaks of AP, which is due to the amorphous structure of estane and therefore does not affect the crystalline phases of AP. Some changes in the intensities of the diffraction peaks, such as 19.4, 23.9°, 24.7°, 30.1°, and 30.9°, can be found in the XRD patterns of the HNS content of 10% and 15% in the Fig. 2. However, the peak positions did not change, which indicates that the crystalline phases of AP did not change during the whole preparation process, mainly since AP was not dissolved during the experiment.

### 3.2. Morphology and structure of AP@HNS composites

**Fig. 3:**
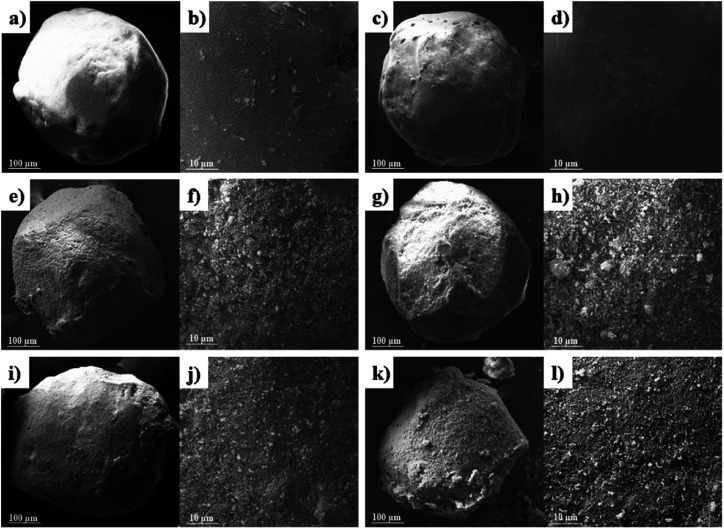
SEM images of the samples: AP (a and b), E-AP (c and d), core–shell AP@HNS composites with 5% (e and f), 10% (g and h), 15% (i and j) and 20% (k and l) HNS content.

The morphology, size, and structure of energy-containing materials are closely related to their safety and thermal properties. Using SEM, this study has characterized the raw AP, modified E-AP, and core–shell AP@HNS composites with different HNS ratios. As shown in Fig. 3(a) and (b), the size of the raw AP particles is around 400 μm, which are sphere-like with smooth and flat surfaces. Fig. 3(c) and (d) shows the E-AP. The particle surface becomes smoother and flatter, with a sphere-like shape, confirming the successful modification of estane on AP particles. Fig. 3(e) and (f) shows the AP@HNS prepared by 5% HNS, from which it is evident that the surface of AP particles becomes rough. It is evident that there are nano-HNS deposited on the surface of AP after magnification, but HNS does not form a complete shell structure on the surface of AP particles. When the content of HNS is 10% (Fig. 3(g) and (h)), the nano-HNS on the surface of AP increases. Still, when the content of HNS was 10% (Fig. 3(g) and (h)), the HNS nanoparticles on the surface of AP increased significantly. Still, they did not thoroughly coat the surface of AP. When the HNS in the AP@HNS composites increased to 15% (Fig. 3(i) and (j)), a complete and homogeneous dense core–shell composites was formed, while the AP@HNS prepared with 20% HNS (Fig. 3(k) and (l)) also formed a complete dense core–shell composites. However, there was a partial accumulation and agglomeration of HNS on the AP particles surface and agglomeration. It can be seen from the above results that all the composites are sphere-like and the morphology does not change compared with the AP feedstock, mainly because the AP crystals were not dissolved during the whole preparation process, with the increase of the shell HNS content, its coverage on the AP surface gradually increases, and this structure promotes a larger contact area between the two, which is conducive to the synergistic effect of thermal decomposition, the spherical morphology and the uniform coverage of the shell layer. The shell layer's spherical shape and uniform coverage with blunt HNS are favorable for enhancing AP's safety performance.

### 3.3. Characterization by XPS analysis

**Fig. 4:**
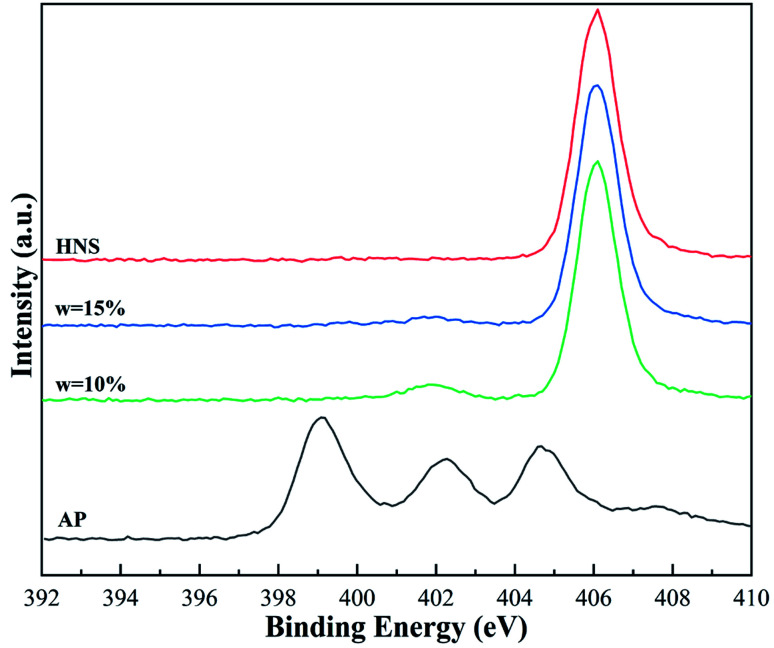
N 1s XPS spectra of AP, nano-HNS, and core–shell AP@HNS with 10% and 15% of HNS.

To further verify the microstructure of AP@HNS, the prepared samples were analyzed and characterized by the XPS test method. The N 1s spectra of the raw AP, core–shell AP@HNS composites with 10% and 15% HNS content, and pure HNS are shown in Fig. 4. It can be seen that although both AP and HNS are composed of H, O, and N elements, the N 1s spectra of AP and HNS are very different. There are three characteristic peaks in the N 1s spectrum of AP with binding energies at 399.1 eV, 402.2 eV, and 404.7 eV, respectively, while there is only one prominent peak in the N 1s spectrum of HNS with a binding energy of 406.1 eV. It was further observed that AP@HNS with 10% HNS content showed two characteristic peaks at 401.9 eV and 406.1 eV. The other two characteristic peaks of AP disappeared, indicating that HNS has a good encapsulation effect. When the shell HNS content of AP@HNS increased to 15%, the characteristic peak at 401.9 eV also disappeared, and only the characteristic peak with the binding energy of 406.1 eV appeared, indicating that the encapsulation rate of HNS reached 100%. These findings are consistent with the above SEM results, thus further confirming the successful preparation of AP@HNS composites with core–shell structure.

### 3.4. Thermal decomposition properties of AP@HNS composites

**Fig. 5:**
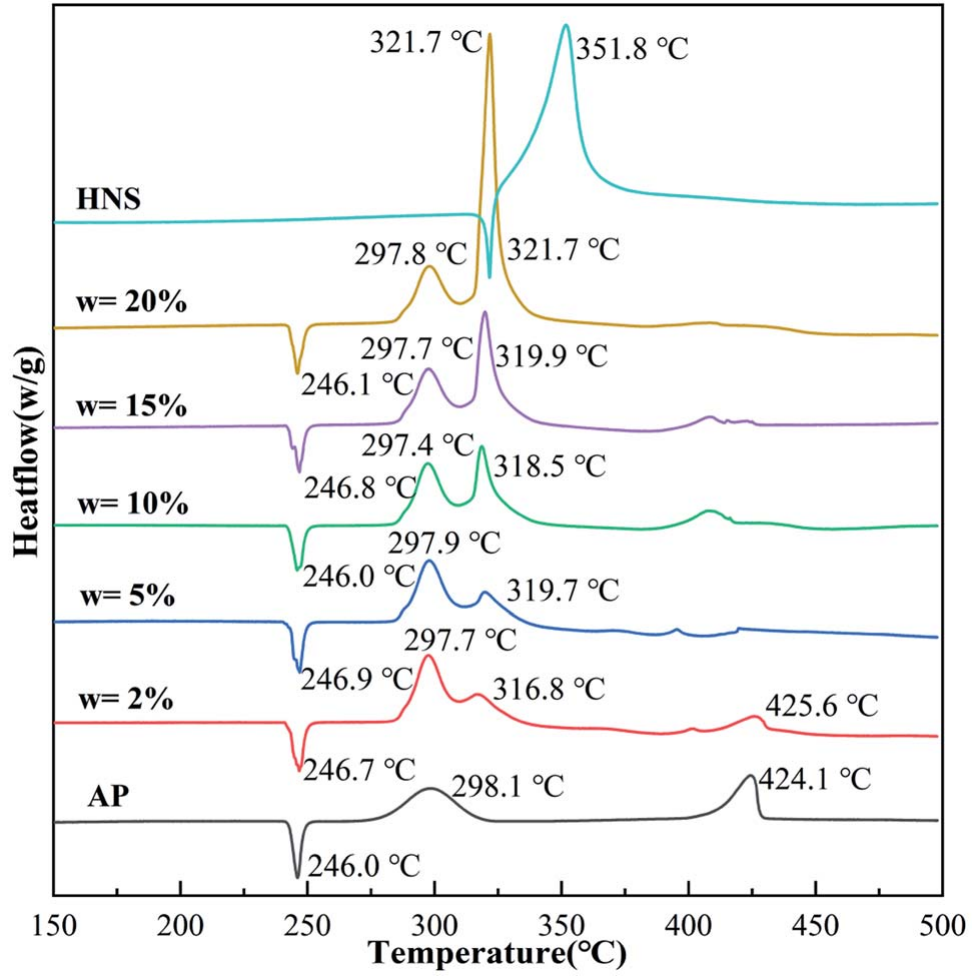
DSC curves of AP, nano-HNS and core–shell AP@HNS with shell HNS content of 2%, 5%, 10%, 15%, and 20%.

The thermal decomposition performance of the composites is an essential parameter for determining their catalytic performance and energy. In order to analyze the thermal decomposition performance of the core–shell AP@HNS composites prepared in this paper, the samples were characterized by DSC analysis, as shown in Fig. 5 and Table 1, which demonstrate the DSC curves and exothermic heats of AP, HNS and core–shell AP@HNS composites with different contents of HNS at a heating rate of 10 K min^−1^. The decomposition of AP is divided into three stages: AP has a heat absorption peak at 246.0 °C, which is caused by the transformation of AP from rhombohedral to cubic crystals. At the following two peaks, located at 298.1 °C and 424.1 °C, AP will decompose from partial thermal decomposition to complete gaseous decomposition and release about 545 J g^−1^ of heat. In addition, the DSC curve of HNS shows that the thermal decomposition process consists of two stages, at 321.7 °C, HNS will melt and absorb some heat, followed by an exothermic decomposition peak at 351.8 °C, which will completely transform into gaseous products with an apparent exothermic heat of 1712 J g^−1^. For the prepared core–shell AP@HNS composites, it can be found that HNS does not affect the transcrystallization temperature of AP. At the same time, it has a significant improvement effect on the thermal decomposition of AP. When the content of shell HNS was 2%, three exothermic peaks of thermal decomposition appeared at 297.7 °C, 316.8 °C, and 425.6 °C, respectively, and it is worth mentioning that the exothermic peak of HNS at 351.8 °C was shifted forward to 319.7 °C, indicating a particular synergistic thermal decomposition effect between HNS and AP. It can be observed that the area of the new exothermic peak at about 320 °C on the DSC curve of the AP@HNS composites gradually increased with the increase of the HNS content, while the area of the decomposition peak at about 400 °C gradually became smaller. When the shell HNS content increased to 20%, the original high-temperature decomposition peak completely disappeared and advanced to 321.7 °C, and its apparent exothermic heat was as high as 1388 J g^−1^. The above data indicate that the higher the HNS content, the more complete the shell is, which increases the contact area between HNS and AP, and the more pronounced the synergistic effect.

**Table 1:** Comparison of the decomposition temperature and heat release of AP, nano-HNS, and core–shell AP@HNS

Sample	HNS (wt%)	LTD (°C)	HTD (°C)	Heat release (J g^−1^)	Increase times
AP	0	298.1	424.1	545	1
w = 2%	2	297.7	425.6	692	1.27
w = 5%	5	297.9	319.7	738	1.35
w = 10%	10	297.4	318.5	865	1.59
w = 15%	15	297.7	319.9	1064	1.95
w = 20%	20	297.8	321.7	1388	2.55
HNS	100	351.8		1712	Sample HNS (wt%)

**Fig. 6:**
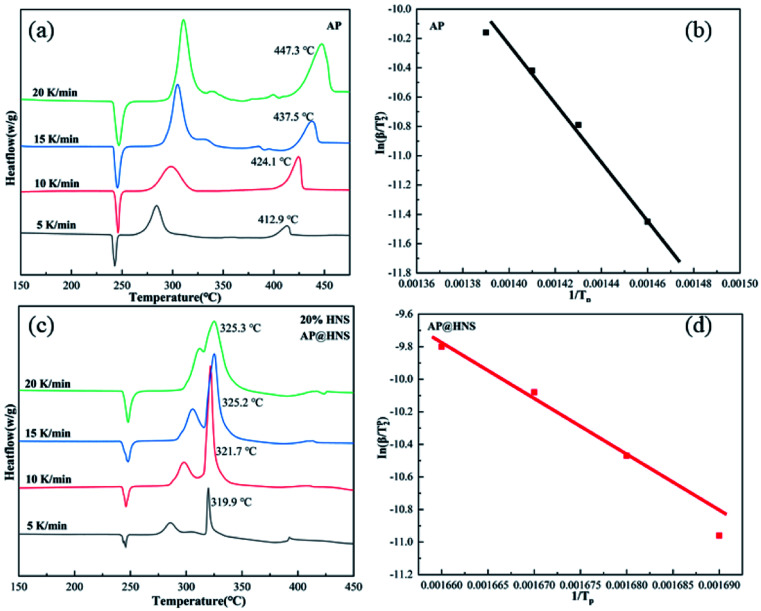
(a) DSC curves of AP at different ramp rates, (b) 1/*T*_p_*vs.* ln(*β*/*T*_p_^2^) straight lines fitted according to AP, (c) DSC curves of AP@HNS at different ramp rates, (d) 1/*T*_p_*vs.* ln(*β*/*T*_p_^2^) straight lines fitted according to AP@HNS.

In order to analyze the effect of nano-HNS on the thermal decomposition process of AP, the activation energy was further introduced to analyze the catalytic performance of HNS. The activation energy is the minimum energy required for the activation molecules to collide effectively and produce a chemical reaction. The concentration of activation molecules determines the rate of this chemical reaction. The DSC curves of AP and the prepared AP@HNS composites were tested at different ramp rates, where the content of HNS was 20%, and the four ramp rates were set at 5 °C min^−1^, 10 °C min^−1^, 15 °C min^−1^, and 20 °C min^−1^, and the activation energies of AP and AP@HNS composites were finally obtained by calculation. For the calculation of the activation energy, this paper is based on the currently widely accepted Kissinger calculation method, with the equations shown below :^22^1
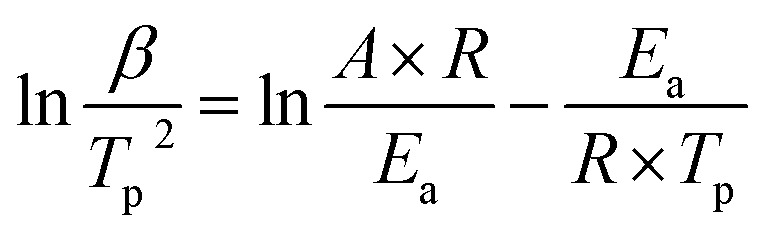
As in the above equation, *β* (°C min^−1^) denotes the heating rate of the measured DSC, *T*_p_ (K) denotes the pyrolysis temperature of the sample, *A* denotes the prefactor, *R* denotes the ideal gas constant of 8.314 J mol^−1^ K^−1^, and *E*_a_ (kJ mol^−1^) denotes the activation energy. To simplify the calculation of *E*_a_, 1/*T*_p_ was set as the horizontal coordinate, and ln(*β*/*T*_p_^2^) was set as the vertical coordinate. The four coordinated points were calculated by fitting a straight line, as shown in Fig. 6(b) and (d). *E*_a_ of AP was calculated to be 168.2 kJ mol^−1^, while *E*_a_ of the AP@HNS composites was 143.7 kJ mol^−1^. It is known that the *E*_a_ of the AP decomposition reaction is related to the HN_3_ and HClO_4_ produced by its initial dissociation. The smaller value of *E*_a_ indicates the higher catalytic activity of the catalyst. Therefore, the significant decrease in *E*_a_ of the AP@HNS composites indicates that HNS and the core–shell structure can effectively improve the kinetics of AP thermal decomposition.

### 3.5. AP@HNS composites susceptibility test

**Fig. 7:**
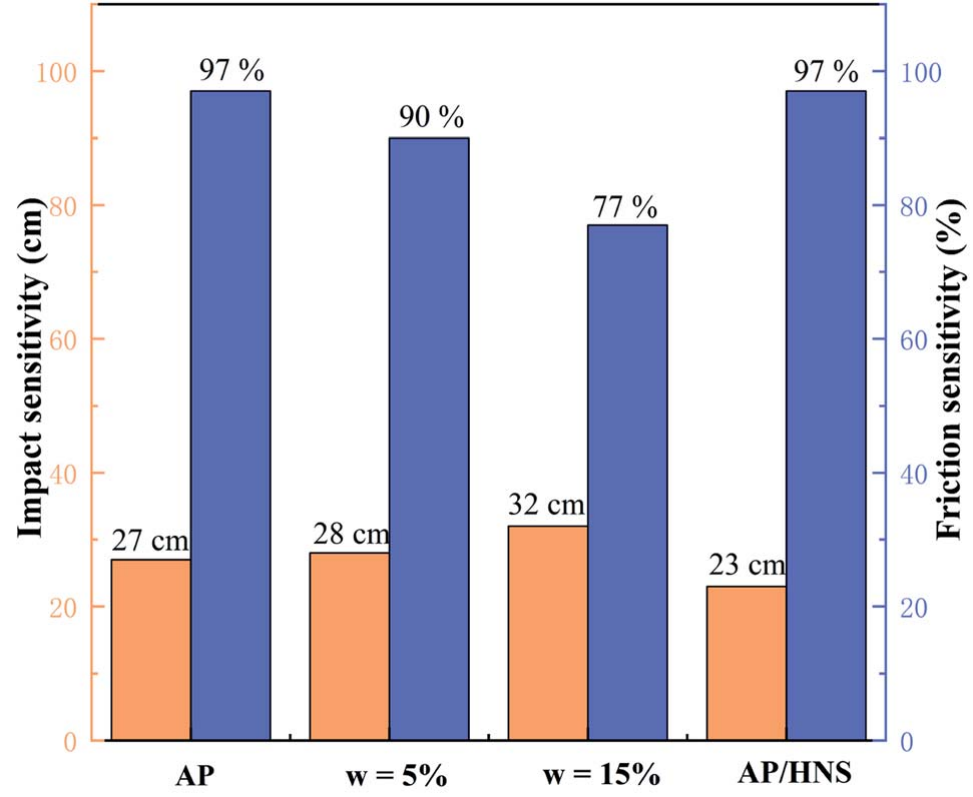
Comparison of impact and friction sensitivities: AP, core–shell AP@HNS composites with 5% and 15% shell content, and mechanically mixed AP/HNS composites with 10% HNS content.

In order to analyze the safety performance of the core–shell AP@HNS composites, the impact susceptibility and friction susceptibility tests were conducted for the core–shell AP@HNS composites with 5% and 15% of HNS as well as mechanically mixed AP/HNS in this paper, and the results are shown in Fig. 7. As shown in the Fig. 7, the *H*_50_ value of the raw AP is 27 cm with 95% probability of an explosion. For the AP@HNS composites with core–shell structure, the impact and frictional sensitivities are somewhat improved, *e.g.*, for AP@HNS with 15% shell content, the *H*_50_ increases to 32 cm, and the probability of explosion decreases to 77%. It is noteworthy that the safety performance of mechanically mixed AP/HNS is reduced, which may be because mechanical mixing results in a much smaller size of AP particles, making it easier to generate hot spots in impact and friction. The shell of the core–shell structure can act as a cushion and lubricant, thus reducing the generation of hot spots and improving the safety of AP. Therefore, the core–shell structure of AP@HNS is an effective way to improve the safety of AP and composites.

### 3.6. AP@HNS synergistic thermal decomposition mechanism

**Fig. 8:**
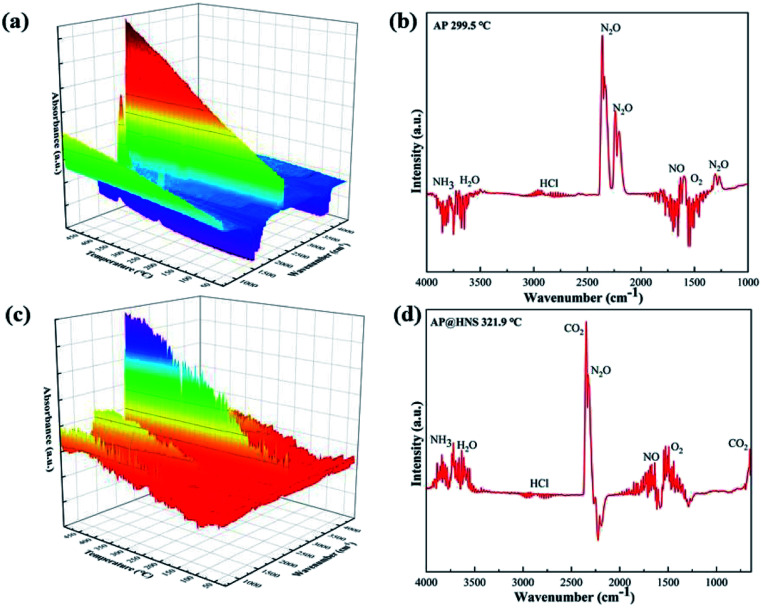
Real-time FT-IR spectra of (a) AP and (c) AP@HNS composites, (b) AP at 299.5 °C and (d) AP@HNS at 321.9 °C.

Our previous work on the synergistic pyrolysis performance of core–shell AP@HNS composites confirmed that the nano-HNS and core–shell structures have a good catalytic effect on AP. For this purpose, the possible catalytic mechanism of HNS on AP was discussed. Real-time FT-IR data of AP and AP@HNS was obtained at a temperature increase rate of 10 °C min^−1^ using the thermal red coupling technique, and their temperature-dependent 3D FT-IR patterns are shown in Fig. 8(a) and (c). The FT-IR patterns of the individual temperature nodes are also shown in Fig. 8(b) and (d). As shown in the Fig. 8, the FT-IR spectra of AP are at 299.5 °C. It can be seen that the decomposition products at this time are mainly NH_3_, H_2_O, HCl, N_2_O, O_2_, and NO, which is consistent with those reported in the literature.^23,24^ However, compared with pure AP, the FT-IR spectrum of the AP@HNS composites at 321.9 °C showed that the products contained more NO gas. The ratio of N_2_O to NO gas was reduced, indicating that the HNS in AP@HNS promoted the oxidation of HN_3_ and accelerated the oxidation of N_2_O to NO (N_2_O + O_2_ → NO), which might be due to the release of the acidic intermediate product-HClO_4_ in the decomposition stage of AP at low temperature. When the content of HClO_4_ reaches a particular concentration, HClO_4_ stimulates the N atom in HNS, which makes HNS decompose early and releases a large amount of NO_2_. Moreover, it reverses the oxidation of NH_3_ produced by AP decomposition, thus advancing the second process of AP thermal decomposition.^25^

## 4. Conclusion

Based on the synergistic effect of AP and explosives, the core–shell structured AP@HNS composites were prepared by the ultrasonic synthesis in this paper by introducing estane as the surface modifier, taking advantage of the high energy and passivity properties of HNS. The summary of this chapter is as follows:

The XRD analysis confirmed that the preparation conditions and the HNS shell structure in this thesis do not change the crystalline phases of AP. The SEM and XPS analyses showed that the AP@HNS composites were prepared with a complete and dense shell structure when the content of shell HNS was above 15%. DSC analysis concluded that the shell HNS could produce a unique synergistic effect with AP, resulting in excellent thermal decomposition properties. For example, the shell HNS content of 20% could advance the high-temperature decomposition peak to 321.7 °C, and its apparent heat release increased by 2.55 times to 1388 J g^−1^. The corresponding core–shell AP@HNS composite showed good safety performance in the susceptibility test. 15% content of HNS can increase the *H*_50_ of AP@HNS composite to 32 cm and reduce the explosion probability to 77%. The preparation technique in this thesis can be used to uniformly coat nano-HNS shells with different types of energy-containing material cores to modulate the safety and thermal decomposition properties. This technique is expected to provide new ideas for designing and preparing solid propellants with high energy and low susceptibility and excellent thermal decomposition properties.

## Conflicts of interest

The authors declare that they have no known competing financial interests or personal relationships that could have appeared to influence the work reported in this paper.

## Supplementary Material
